# Volume of the right supramarginal gyrus is associated with a maintenance of emotion recognition ability

**DOI:** 10.1371/journal.pone.0254623

**Published:** 2021-07-22

**Authors:** Sayaka Wada, Motoyasu Honma, Yuri Masaoka, Masaki Yoshida, Nobuyoshi Koiwa, Haruko Sugiyama, Natsuko Iizuka, Satomi Kubota, Yumika Kokudai, Akira Yoshikawa, Shotaro Kamijo, Sawa Kamimura, Masahiro Ida, Kenjiro Ono, Hidetoshi Onda, Masahiko Izumizaki

**Affiliations:** 1 Department of Physiology, Showa University School of Medicine, Tokyo, Japan; 2 Department of Ophthalmology, Jikei University School of Medicine, Tokyo, Japan; 3 Human Arts and Sciences Research Center, University of Human Arts and Sciences, Saitama, Japan; 4 Sensory Science Research, Kao Corporation, Tokyo, Japan; 5 Division of Neurology, Department of Medicine, Showa University School of Medicine, Tokyo, Japan; 6 Department of Radiology, Stroke Center, Ebara Tokyo Hospital, Tokyo, Japan; 7 Department of Ophthalmology, Showa University School of Medicine, Tokyo, Japan; Medical University of Vienna, Austria, AUSTRIA

## Abstract

Emotion recognition is known to change with age, but associations between the change and brain atrophy are not well understood. In the current study atrophied brain regions associated with emotion recognition were investigated in elderly and younger participants. Group comparison showed no difference in emotion recognition score, while the score was associated with years of education, not age. We measured the gray matter volume of 18 regions of interest including the bilateral precuneus, supramarginal gyrus, orbital gyrus, straight gyrus, superior temporal sulcus, inferior frontal gyrus, insular cortex, amygdala, and hippocampus, which have been associated with social function and emotion recognition. Brain reductions were observed in elderly group except left inferior frontal gyrus, left straight gyrus, right orbital gyrus, right inferior frontal gyrus, and right supramarginal gyrus. Path analysis was performed using the following variables: age, years of education, emotion recognition score, and the 5 regions that were not different between the groups. The analysis revealed that years of education were associated with volumes of the right orbital gyrus, right inferior frontal gyrus, and right supramarginal gyrus. Furthermore, the right supramarginal gyrus volume was associated with the emotion recognition score. These results suggest that the amount of education received contributes to maintain the right supramarginal gyrus volume, and indirectly affects emotion recognition ability.

## Introduction

The ability to infer the emotions of others is an integral component of social interaction, and an important function that facilitates social communication. Several brain mechanisms are known to be involved in emotion recognition. Some functional magnetic resonance imaging (fMRI) studies in healthy younger persons have shown that emotion recognition is subserved by the superior temporal sulcus [1], inferior frontal gyrus [2], orbitofrontal cortex [3], insula cortex [4], and amygdala/hippocampus [5]. Furthermore, a voxel-based morphometry study on structural MRI data in healthy younger people reported that the gray matter volume of the right inferior frontal gyrus was positively correlated with the accuracy of emotion recognition [6]. In particular, the orbitofrontal cortex is activated by emotional faces rather than neutral faces [7, 8], which suggests that the orbitofrontal cortex is engaged when judging the emotions of others from a picture of a face.

The ability to recognize facial expressions changes with age [9–14]. Although it has been noted that the effects of aging are different for each emotion expression, these results have not been consistent. For example, while it has been reported that elderly adults have the greatest difficulty in recognizing emotions of anger, fear, sadness, and happiness, and that there are no age-related difference in disgust [15], no difference in the recognition of happiness between elderly and younger adults has also been found [16]. These contrasting results may be because various factors can impact emotion recognition. For example, education is thought to be closely related to cognitive ability. Indeed, some meta-analyses have suggested that the number of years of education is a protective factor against cognitive decline and dementia [17, 18]. Furthermore, a previous study reported that individuals with less education are associated with stronger amygdala responses to emotional stimuli, suggesting that educational experience influences brain activity to facial expression stimuli [19].

The definition of emotion is intricate and includes at least the aspects of complexity (basic vs. social), arousal (excited vs. calm), and valence (positive vs. negative) [20, 21]. In the present study, we limited to facial expressions corresponding to the basic emotions (example, Ekman’s study [22]), and used four expressions (angry, sad, happy, and surprised) including arousal and valence. We hypothesized that the emotion recognition may be related to aging and education, and that there may be some brain regions that could account for this relationship. The current study measured gray matter volume of structural MRI data and implemented an emotion recognition test. We performed a region-of-interest (ROI) analysis based on previous research related with social functions, such as empathy (the precuneus) [23], theory of mind (the supramarginal gyrus) [24, 25], interpersonal perception (the ventral frontal cortex, consisting of the orbitofrontal cortex and straight gyrus) [26, 27], and emotion recognition (the superior temporal sulcus [1], inferior frontal gyrus [2, 6], orbitofrontal cortex [3, 7, 8], insular cortex [4], amygdala, and hippocampus [5]). The total 18 locations (on the left and right side of the ROIs) were compared in elderly subjects and younger subjects to identify atrophied regions. Furthermore, we identified the regions responsible for emotion recognition from the perspective of age and education.

## Materials and methods

### Experimental design and participants

Subjects included in this study were a subset of subjects from a previously published study [28], for whom both facial expression and structural MRI data were available. This study was approved by the Ethics Committee of Showa University Hospital and was conducted in accordance with the principles of the Declaration of Helsinki (clinical trial identifier number: 2561). The criteria for age was 23 to 59 years in the younger group, and from 60 to 85 years in the elderly group. All participants provided written informed consent. Twenty elderly participants (10 men and 10 women) and 20 younger adults (11 women and 9 men) participated in this study. Cerebral infarction was diagnosed in one man in the elderly group, and his data were excluded from the analysis. 19 elderly and 18 younger participants were right-hand dominant, and all had normal visual acuity. The average age in the elderly group (mean age: 74.1 years; range: 68 to 82) was higher than that in younger group (mean age: 40.7 years; range: 24 to 59) (*t*_37_ = 11.970, *p* < 0.0001). The average number of years of education in the elderly group was lower than that in younger group (*t*_37_ = 4.610, *p* < 0.0001). The average Montreal Cognitive Assessment (MoCA) score [29] in the elderly group was lower than that in younger group (*t*_37_ = 3.012, *p* = 0.005) (Table 1).

**Table 1:** Participants details.

		Younger group		Elderly group		*p* value
		Average	S.D.	Average	S.D.	
Age		40.7	11.5	74.1	4.3	< 0.0001
Sex						
	Female	11		10		
	Male	9		9		
MoCA		27.9	2.1	25.8	2.2	0.005
Years of Education		17.1	2.4	13.4	2.6	< 0.0001

MoCA: Montreal Cognitive Assessment (max: 30). Years of Education: Years of education since entering elementary school.

### Emotion recognition test

**Fig 1:**
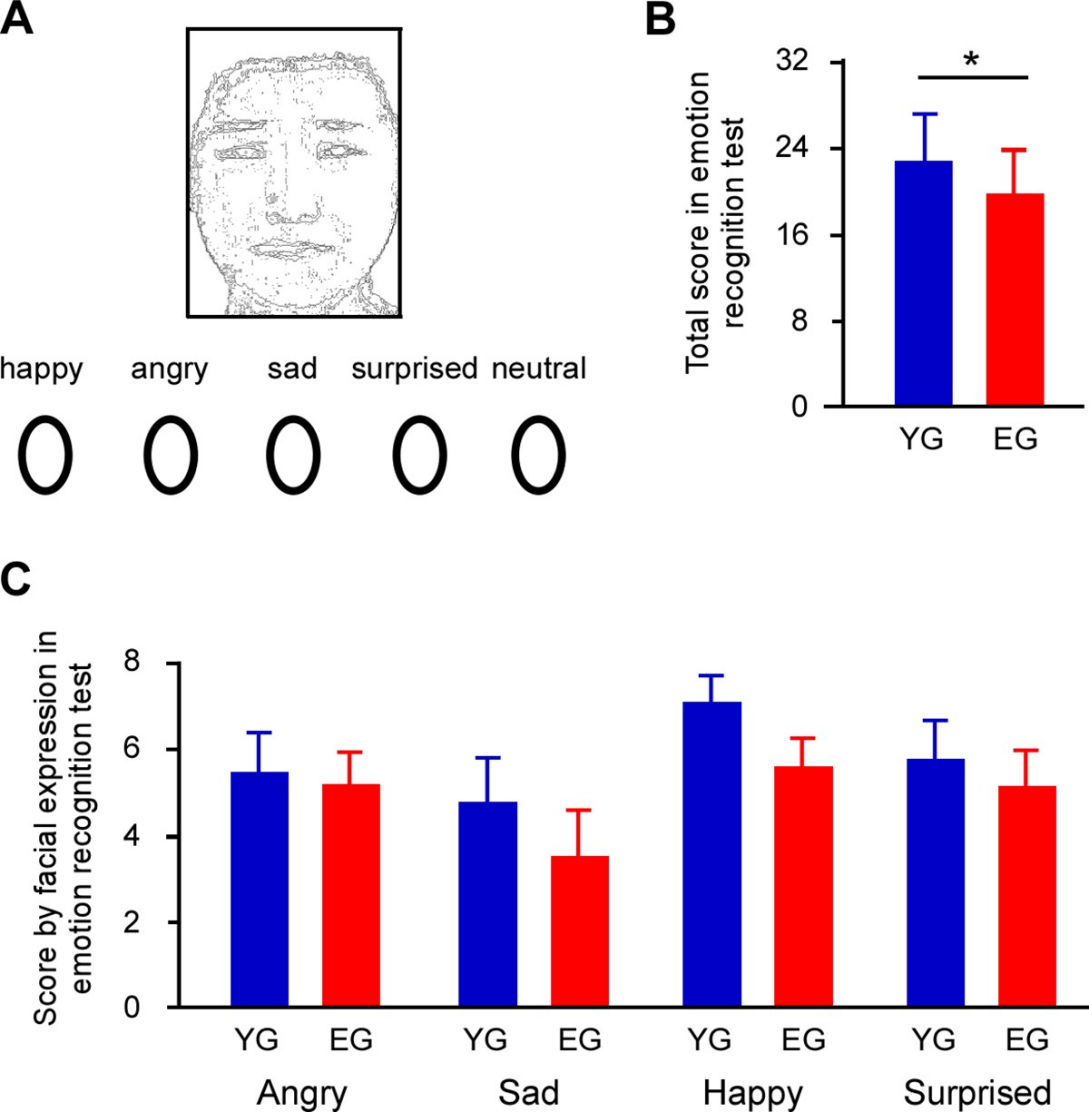
Protocol and results of the emotion recognition test. **(A)** A representation of a trial. One image of four facial expressions (angry, sad, happy, or surprised) was presented, and the participant was asked to choose a facial expression from five options (angry, sad, happy, surprised, and neutral). The facial picture is illustrated in this paper. **(B)** The *t*-test revealed that the total emotion recognition score in the elderly group (EG) was lower than that of younger group (YG). **(C)** An RM-ANCOVA with years of education and MoCA scores as covariates showed that there was no main effect of facial expression or group, and no interaction. Asterisks indicate significant differences (*p* < 0.05). Error bars indicate the standard error of mean.

Facial expressions were performed by 2 Japanese men and 2 Japanese women who were not known to the participants prior to the study. The 4 different facial expressions were used–angry, sad, happy, and surprised. Facial images were created using morphing techniques, whereby a neutral expression was merged with the given facial expression, to increase task difficulty. Participants were asked to choose a facial expression from 5 options (angry, sad, happy, surprised, or neutral) while looking at a presented face (Fig 1A). The 32 trials (8 images for each facial expression) were conducted by each participant (maximum score 32).

### MRI acquisition

MRI scanning was performed at Ebara Hospital using a Siemens Avanto 3 T Magnetom TIM Trio scanner. T1-weighted images were acquired using an optimized Magnetization-Prepared Rapid Acquisition Gradient Echo sequence. T1-weighted high-resolution anatomical images were obtained using a magnetization-prepared rapid gradient-echo sequence (repetition time 2250 ms, echo time 3.06 ms, flip angle 9 degrees, inversion time 1000 ms, field of view 256 x 256 mm, matrix size 256 x 256, voxel size 1 x 1 x 1 mm).

### Image processing

Intracranial volumes (ICVs) were estimated using FreeSurfer version 6 [30, 31], which uses an atlas scaling factor (i.e., the determinant of an affine transformation matrix) derived by registering images to an Montreal Neurological Institute (MNI) space using a full 12-parameter affine transformation [32], followed by removal of the skull and other non-brain tissue from the pial and white matter surfaces for each subject, which were spherically inflated and registered to the manually delineated Desikan-Killiany brain atlas [33]. Data pre-processing was inspected to confirm successful registration to the MNI space, and all boundaries were visually inspected and manually corrected to produce accurate estimates.

The 70 anatomical brain areas were automatically labeled according to sulco-gyral structures using the FreeSurfer recon-all stream. Detailed descriptions have been provided elsewhere [34]. In brief, the nomenclature used in FreeSurfer saved as aparc.aseg files was based on those of Duvernoy’s study [35], which indicated that classical anatomical nomenclature covers the Talairach structural parcellations [36]. Classical anatomical atlases were used to identify brain regions according to gyral classification [35, 36]. The disadvantage of gyrus classification is that gyral labels extend to the border of the sulci. To solve this issue, sulco-gyral-based parcellation has been proposed, whereby the gyral cortex was defined as the one seen on a 3D reconstruction before inflation (pial view), the remaining hidden part being conversely labeled sulcal [36].

### Statistical analysis

An unpaired *t*-test was used to compare the total emotion recognition score, age, years of education, and MoCA score between the two groups. A two-way repeated measures analysis of covariance (RM-ANCOVA) was used to assess the relationship between facial expressions (angry, sad, happy, and surprised) and groups (younger and elderly), with years of education and MoCA scores as covariates. Brain volumes were divided into 70 regions, and 18 regions were selected as ROIs. An ANCOVA was used to compare the region volumes between groups, with ICVs, years of education, and MoCA scores as covariates. Post-hoc *t*-tests without Bonferroni correction were performed for multiple comparisons in RM-ANCOVA and ANCOVA. The binomial test was used to examine the male-female ratio of samples. All tests were two-tailed. The results are shown as the mean ± the standard error of the mean. Stepwise multiple regression was used to assess associations between the emotion recognition test score (independent variable) and age, years of education, and MoCA score (dependent variables). SPSS version 26 was used for these statistical analyses. Relationships between the years of education, some regions, and emotion recognition score were determined using path analysis. The goodness of fit of index, direct effect, and indirect effect were calculated. AMOS 27.0 was used for path analysis. Statistical significance was defined as an adjusted *p* < 0.05.

## Results

The binomial test showed that the male-female ratio of samples was not different between groups (*p* = 0.749). The Kolmogorov-Smirnov test of normality showed that total score of emotion recognition test was normally distributed within both younger and elderly groups (both, *p* = 0.200). The normality for the facial expressions in the younger group were as follows: angry, *p* = 0.130; sad, *p* = 0.200; happy, *p* = 0.064; and surprised, *p* = 0.044. The normality for the facial expressions in the elderly group were as follows: angry, *p* = 0.012; sad, *p* = 0.200; happy, *p* = 0.001; and surprised, *p* = 0.059. The total emotion recognition score was significantly lower in the elderly group than in the younger group (unpaired *t*-test, t_37_ = 2.475, *p* = 0.018; Fig 1B). For the 4 facial expressions (Fig 1C), the two-way RM-ANCOVA with years of education and MoCA scores as covariates revealed that there was no main effect of facial expression, (*F*_3,102_ = 1.007, *p* = 0.393, *η*^2^ = 0.029), no effect of group (*F*_1,34_ = 0.028, *p* = 0.867, *η*^2^ = 0.001), and no significant interaction between facial expression and group (*F*_3,102_ = 0.334, *p* = 0.801, *η*^2^ = 0.010). However, multiple regression analysis revealed that years of education (and not age or MoCA score) were associated with emotion recognition score (*B* = 0.534, *p* < 0.0001).

The ANCOVA with ICVs, years of education, and MoCA scores as covariates and uncorrected multiple comparisons revealed that 13 of the 18 regions differed significantly between the age groups (Table 2). The elderly group exhibited smaller volumes in the left insula (*p* = 0.001), left orbital gyrus (*p* = 0.001), left supramarginal gyrus (*p* = 0.002), left precuneus (*p* = 0.044), left superior temporal gyrus (*p* = 0.015), right inferior frontal gyrus (*p* = 0.011), left hippocampus (*p* < 0.0001), left amygdala (*p* < 0.0001), right precuneus (*p* = 0.039), right straight gyrus (*p* = 0.007), right superior temporal gyrus (*p* = 0.005), right hippocampus (*p* < 0.0001), and right amygdala (*p* < 0.0001) than the younger group.

**Table 2:** Group differences in volumes.

Region	Younger group		Elderly group		*p* value
	Average	S.D.	Average	S.D.	
left inferior frontal gyrus	1098.6	164.2	994.5	197.7	0.122
left insula gyrus	1602.0	194.0	1291.7	204.4	0.001
left orbital gyrus	5927.2	628.4	5001.0	655.0	0.001
left supramarginal gyrus	7061.7	1122.3	5704.2	835.5	0.002
left precuneus	6231.1	854.5	5386.9	905.3	0.044
left straight gyrus	2271.9	305.3	2159.8	253.9	0.835
left superior temporal gyrus	5927.9	735.6	5132.5	767.1	0.015
left hippocampus	4171.6	350.3	3439.8	348.6	< 0.0001
left amygdala	1773.5	171.0	1350.1	174.6	< 0.0001
right inferior frontal gyrus	1030.8	193.7	865.6	202.9	0.323
right insula gyrus	1634.7	253.4	1331.8	134.1	0.011
right orbital gyrus	6670.6	741.8	5891.4	609.7	0.063
right supramarginal gyrus	6241.0	1319.9	5032.1	572.3	0.106
right precuneus	5700.8	1000.3	4913.1	889.4	0.039
right straight gyrus	2072.0	261.6	1742.3	191.9	0.007
right superior temporal gyrus	5126.1	657.8	4369.4	724.7	0.005
right hippocampus	4389.3	324.1	3608.9	427.7	< 0.0001
right amygdala	1822.2	154.8	1521.4	189.7	< 0.0001

**Fig 2:**
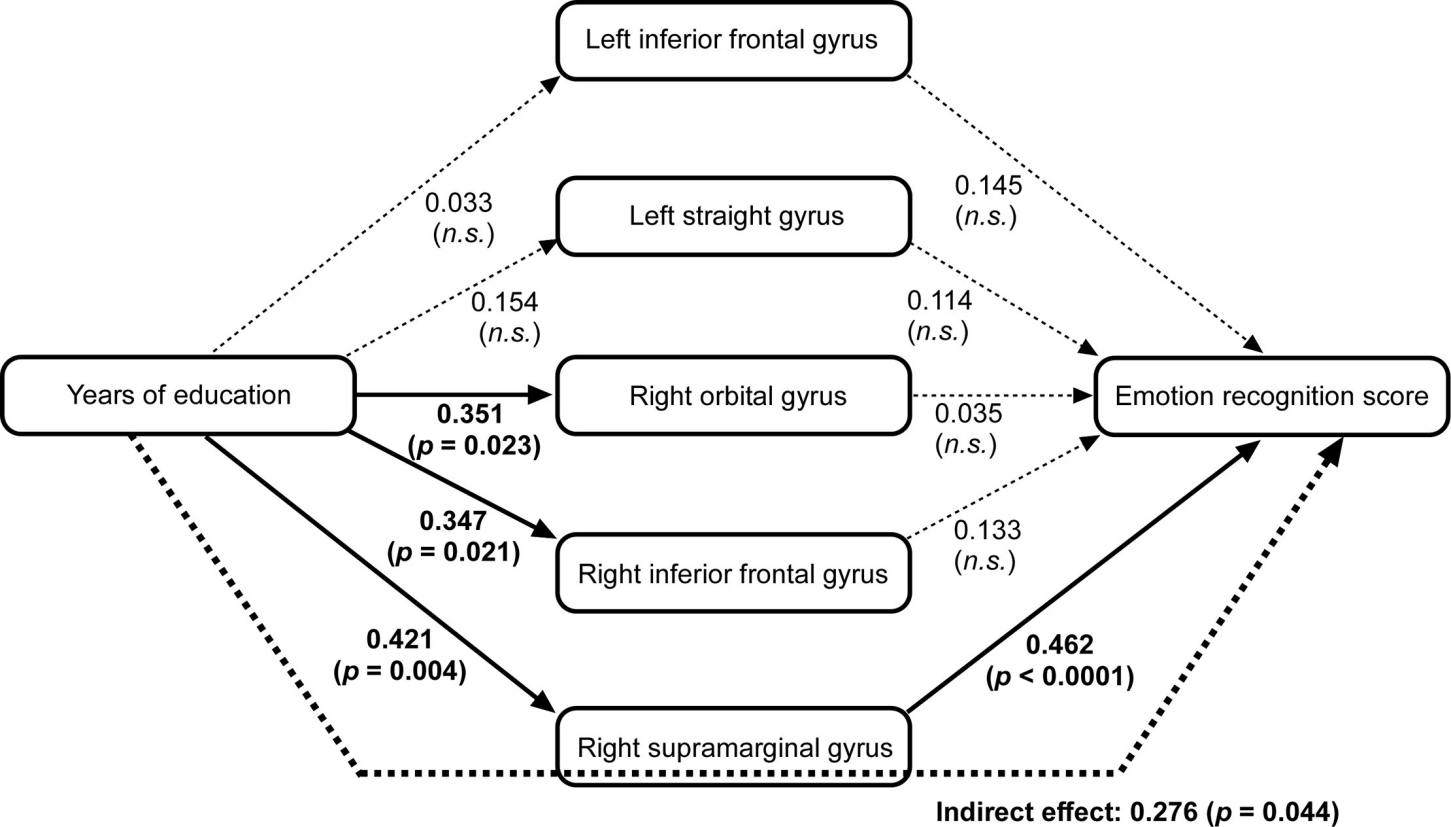
Path diagram. The 5 regions, years of education, and emotion recognition score were set as the observed variables. The solid lines indicate a significant direct effect. The dotted thick line indicates a significant indirect effect. Thin dotted lines indicate a non-significant direct path. Numbers indicate the standardized path coefficients.

We conducted a path analysis to examine a mechanistic route for the emotion recognition results. We focused on the 5 regions within which there were no significant between-group differences (the left inferior frontal gyrus, left straight gyrus, right orbital gyrus, right inferior frontal gyrus, and right supramarginal gyrus) to identify the protective factors against a change in emotion recognition. The 5 regions, years of education, and emotion recognition score were set as the observed variables. Years of education were associated with the volume of right orbital gyrus (*p* = 0.023), right inferior frontal gyrus (*p* = 0.021), and right supramarginal gyrus (*p* = 0.004), and the right supramarginal gyrus was significantly associated with the emotion recognition score (*p* < 0.0001). The most suitable model was the route from years of education to emotion recognition score via the right supramarginal gyrus (goodness of fit of index = 0.907; Fig 2), and the indirect effect between years of education and emotion recognition score was significant (*p* = 0.044)

## Discussion

In the present study, emotion recognition was no different between the elderly and younger groups, and was associated with years of education, but not age or MoCA score. An absence of atrophy was seen in 5 of the 18 brain regions in the elderly group, and the years of education affected three of these–the right orbital gyrus, right inferior frontal gyrus, and right supramarginal gyrus. Furthermore, only the right supramarginal gyrus was associated with emotion recognition. These results suggest that long-term education period may protect against brain atrophy of some regions, and that a preserved right supramarginal gyrus volume may be linked to maintained emotion recognition function.

Although the between-group comparison revealed differences in the total emotion recognition score, the multiple regression analysis showed that age had no effect on the score; only years of education were an important factor. Furthermore, the ANCOVA, which used the type of facial expressions as a factor, showed no difference among facial expressions or between groups in the emotion recognition score. This result may reflect the use of years of education as a covariate. Some previous studies have shown differences between facial expressions, and some have not [15, 16]. Furthermore, previous studies have shown that education period significantly impacts cognitive ability [17, 18], whereby years of education had a strong impact on emotion recognition scores in the current study. This result may have something to do with the educational environment in Japan. While the compulsory education period is short, a high percentage of students go on to university or graduate school in Japan, so the years of education are likely to vary. In addition, the difficulty level of the emotion recognition test may affect the current results. Ekman’s study employed a method in which several photographs of “clear” facial expressions were shown to the participants, and then they were asked which emotion each individual expression represented [22]. On the other hand, in this study, the difficulty level of the facial expression recognition test was increased by morphing images so that even healthy subjects could not get a perfect score. For this reason, the effects of aging became apparent, and we speculated that this was the result of education affecting the scores of the emotion recognition test.

**Fig 3:**
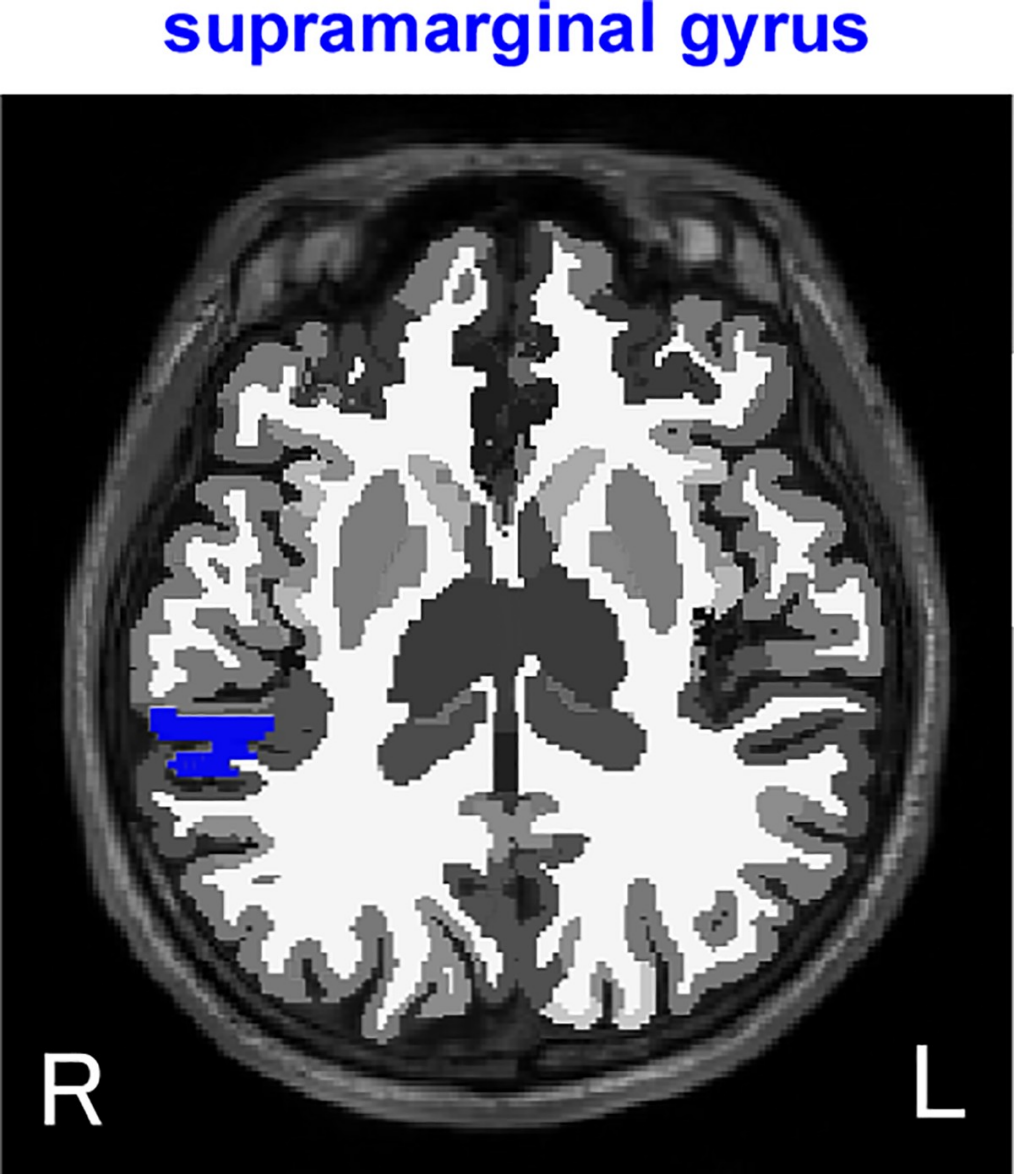
Location of the right supramarginal gyrus in axial view (blue area).

Furthermore, the present results indicate that the right supramarginal gyrus is involved in the relationship between education and emotion recognition. The supramarginal gyrus surrounds the terminal portion of the ascending branch of the lateral sulcus and, together with the angular gyrus, constitutes the inferior parietal lobule (Fig 3). Many studies have reported that the right supramarginal gyrus is associated with Theory of Mind or empathy [37, 38]. Another study also found that this region is related to understanding another person’s internal state [39]. Furthermore, abnormal activity of this region has been associated with a low capacity for sympathy, empathy, and language in autism spectrum disorder [40, 41]. Thus, the right supramarginal gyrus may play a role in social communication. These previous findings support the implications of the present study that maintenance of the volume of the right supramarginal gyrus is related to the maintenance of emotion recognition ability.

The fact that we found no relationship between the left supramarginal gyrus and emotion recognition may be related to a hemispherical specialization of brain. For example, the left supramarginal gyrus has been implicated in tool use and language skills [42, 43]. Thus, the role of the supramarginal gyrus is very different between the left and right hemispheres. It is also interesting to note that the years of education had a non-significant relationship with the left supramarginal gyrus. The current results suggest that the protective effect of education on brain atrophy may be related only to the right (and not the left) supramarginal gyrus. Thus, the effect of education on atrophy may differ between the right and left sides of the same gyrus; our results suggest that education maintains the right supramarginal gyrus, which was associated with the retention of emotion cognitive ability.

The current study has several limitations. First, the emotion recognition test used in this study included neutral expression face in the choices, but not in the stimuli. The presence of a neutral expression is important because it allows for a dichotomous analysis of whether a face contains emotion or not. Second, this study did not take into account the own-age bias, in which face recognition is typically superior for own-age faces compared with other-age faces [44]. In this study, all participants were shown the same photographs of young people 20–30 years of age. The inclusion of the own-age bias factor in the experiment may have also changed the scores on the facial expression recognition test. In the future, it may be necessary to consider the age factor of the facial expression stimuli. Third, the current results cannot be generalized to all cultures and races, because Japanese participants responded to Japanese facial expression stimuli in this experiment. There are reports that different cultures and races tend to read emotions differently [45, 46]. It is therefore important to conduct cross-cultural research. Finally, the current study had a small sample size, in particular, the normality of each facial expression was insufficient. Further research with a larger sample is required to verify the reproducibility of our results. However, this study found that a specific brain region mediates the relationship between emotion recognition and education, which offers valuable insights into the effects of aging.

The current study found a relationship between years of education and emotion recognition ability, which was mediated by the right supramarginal gyrus. With regard to individual differences, emotion recognition function may be preserved in the absence of an atrophied right supramarginal gyrus based on the years of education, regardless of age. However, this study dealt with basic emotions, and the effects of aging and education may be different for higher-order social emotions such as jealousy, embarrassment, guilt, and shame. Although the role of the right supramarginal gyrus remains to be characterized, there is a need to examine the influence of the gyrus on higher-order social communication.

## Supporting information

S1 Data (XLSX)

## References

[1] Narumoto J, Okada T, Sadato N, Fukui K, Yonekura Y. Attention to emotion modulates fMRI activity in human right superior temporal sulcus. Brain Res Cogn Brain Res. 2001;12(2):225–31. Epub 2001/10/06. doi: 10.1016/s0926-6410(01)00053-2 11587892

[2] Sato W, Kochiyama T, Yoshikawa S, Naito E, Matsumura M. Enhanced neural activity in response to dynamic facial expressions of emotion: an fMRI study. Brain Res Cogn Brain Res. 2004;20(1):81–91. Epub 2004/05/08. doi: 10.1016/j.cogbrainres.2004.01.008 15130592

[3] Blair RJ, Morris JS, Frith CD, Perrett DI, Dolan RJ. Dissociable neural responses to facial expressions of sadness and anger. Brain. 1999;122 (Pt 5):883–93. Epub 1999/06/04. doi: 10.1093/brain/122.5.883 10355673

[4] Phillips ML, Young AW, Senior C, Brammer M, Andrew C, Calder AJ, et al. A specific neural substrate for perceiving facial expressions of disgust. Nature. 1997;389(6650):495–8. Epub 1997/10/23 22:27. doi: 10.1038/39051 9333238

[5] Williams LM, Phillips ML, Brammer MJ, Skerrett D, Lagopoulos J, Rennie C, et al. Arousal dissociates amygdala and hippocampal fear responses: evidence from simultaneous fMRI and skin conductance recording. Neuroimage. 2001;14(5):1070–9. Epub 2001/11/08. doi: 10.1006/nimg.2001.0904 11697938

[6] Uono S, Sato W, Kochiyama T, Sawada R, Kubota Y, Yoshimura S, et al. Neural substrates of the ability to recognize facial expressions: a voxel-based morphometry study. Soc Cogn Affect Neurosci. 2017;12(3):487–95. Epub 2016/09/28. doi: 10.1093/scan/nsw142 27672176 PMC5390731

[7] Paulmann Silke, Seifert Sebastian, Sonja A Kotz. Orbito-frontal lesions cause impairment during late but not early emotional prosodic processing. Soc Neurosci. 2010;5(1):59–75. doi: 10.1080/17470910903135668 19658025

[8] Campanella Salvatore, Bourguignon Mathieu, Peigneux Philippe, Metens Thierry, Nouali Mustapha, Goldman Serge, et al. BOLD response to deviant face detection informed by P300 event-related potential parameters: a simultaneous ERP-fMRI study. Neuroimage. 2013 May 1;71:92–103. doi: 10.1016/j.neuroimage.2012.12.077 23313569

[9] Mather M, Carstensen LL. Aging and attentional biases for emotional faces. Psychol Sci. 2003;14(5):409–15. Epub 2003/08/22. doi: 10.1111/1467-9280.01455 12930469

[10] Krendl AC, Ambady N. Older adults’ decoding of emotions: role of dynamic versus static cues and age-related cognitive decline. Psychol Aging. 2010;25(4):788–93. Epub 2010/12/29. doi: 10.1037/a0020607 21186915

[11] Isaacowitz DM, Stanley JT. Bringing an Ecological Perspective to the Study of Aging and Recognition of Emotional Facial Expressions: Past, Current, and Future Methods. J Nonverbal Behav. 2011;35(4):261–78. Epub 2011/11/30. doi: 10.1007/s10919-011-0113-6 22125354 PMC3223963

[12] Ruffman T. Ecological Validity and Age-Related Change in Emotion Recognition. J Nonverbal Behav. 2011;35:297–304. doi: 10.1007/s10919-011-0116-3

[13] Schlegel K, Grandjean D, Scherer KR. Introducing the Geneva emotion recognition test: an example of Rasch-based test development. Psychol Assess. 2014;26(2):666–72. Epub 2013/12/04. doi: 10.1037/a0035246 24295238

[14] Ruffman T, Henry JD, Livingstone V, Phillips LH. A meta-analytic review of emotion recognition and aging: implications for neuropsychological models of aging. Neurosci Biobehav Rev. 2008;32(4):863–81. Epub 2008/02/16. doi: 10.1016/j.neubiorev.2008.01.001 18276008

[15] Darowski ES, Helder E, Zacks RT, Hasher L, Hambrick DZ. Age-related differences in cognition: the role of distraction control. Neuropsychology. 2008;22(5):638–44. Epub 2008/09/04. doi: 10.1037/0894-4105.22.5.638 18763883

[16] Svärd J, Wiens S, Fischer H. Superior recognition performance for happy masked and unmasked faces in both younger and older adults. Front Psychol. 2012;3:520. Epub 2012/12/12. doi: 10.3389/fpsyg.2012.00520 23226135 PMC3510469

[17] Alvares Pereira G, Silva Nunes MV, Alzola P, Contador I. Cognitive reserve and brain maintenance in aging and dementia: An integrative review. Appl Neuropsychol Adult. 2021:1–11. Epub 2021/01/26. doi: 10.1080/23279095.2021.1872079 33492168

[18] Meng X, D’Arcy C. Education and dementia in the context of the cognitive reserve hypothesis: a systematic review with meta-analyses and qualitative analyses. PLoS One. 2012;7(6):e38268. Epub 2012/06/08. doi: 10.1371/journal.pone.0038268 22675535 PMC3366926

[19] Demenescu Liliana R, Stan Adrian, Kortekaas Rudie, van der Wee Nic J A, Veltman Dick J, Aleman André. On the connection between level of education and the neural circuitry of emotion perception. Front Hum Neurosci. 2014 Oct 27;8:866. doi: 10.3389/fnhum.2014.00866 25386133 PMC4209829

[20] José M Mestre Cristina Larrán, Herrero Joaquín, Guil Rocío, de la Torre Gabriel G. PERVALE-S: a new cognitive task to assess deaf people’s ability to perceive basic and social emotions. Front Psychol. 2015 Aug 7;6:1148. doi: 10.3389/fpsyg.2015.01148 eCollection 2015. 26300828 PMC4528103

[21] Satpute Ajay B, Lindquist Kristen A. The Default Mode Network’s Role in Discrete Emotion. Trends Cogn Sci. 2019 Oct;23(10):851–864. doi: 10.1016/j.tics.2019.07.003 Epub 2019 Aug 16. 31427147 PMC7281778

[22] Ekman P., and Friesen W. V. (1971). Constants across cultures in the face and emotion. J. Pers. Soc. Psychol. 17, 124–129. doi: 10.1037/h0030377 5542557

[23] Wrege JS, Ruocco AC, Carcone D, Lang UE, Lee ACH, Walter M. Facial Emotion Perception in Borderline Personality Disorder: Differential Neural Activation to Ambiguous and Threatening Expressions and Links to Impairments in Self and Interpersonal Functioning. J Affect Disord. 2021;284:126–35. Epub 2021/02/17. doi: 10.1016/j.jad.2021.01.042 33592431

[24] Paul S, Arora A, Midha R, Vu D, Roy PK, Belmonte MK. Autistic traits and individual brain differences: functional network efficiency reflects attentional and social impairments, structural nodal efficiencies index systemising and theory-of-mind skills. Mol Autism. 2021;12(1):3. Epub 2021/01/23. doi: 10.1186/s13229-020-00377-8 33478557 PMC7818759

[25] Schurz M, Tholen MG, Perner J, Mars RB, Sallet J. Specifying the brain anatomy underlying temporo-parietal junction activations for theory of mind: A review using probabilistic atlases from different imaging modalities. Hum Brain Mapp. 2017;38(9):4788–805. Epub 2017/06/14. doi: 10.1002/hbm.23675 28608647 PMC6867045

[26] Wood JL, Heitmiller D, Andreasen NC, Nopoulos P. Morphology of the ventral frontal cortex: relationship to femininity and social cognition. Cereb Cortex. 2008;18(3):534–40. Epub 2007/06/19. doi: 10.1093/cercor/bhm079 17573374

[27] Zhen S, Yu R. Neural correlates of recursive thinking during interpersonal strategic interactions. Hum Brain Mapp. 2021. Epub 2021/01/30. doi: 10.1002/hbm.25355 33512053 PMC8046141

[28] Kubota S, Masaoka Y, Sugiyama H, Yoshida M, Yoshikawa A, Koiwa N, et al. Hippocampus and parahippocampus volume reduction associated with impaired olfactory abilities in subjects without evidence of cognitive decline. Frontiers in Human Neuroscience. 2020; 14:556519. doi: 10.3389/fnhum.2020.556519 33192392 PMC7556227

[29] Rossetti HC, Lacritz LH, Cullum CM, Weiner MF. Normative data for the Montreal Cognitive Assessment (MoCA) in a population-based sample. Neurology. 2011;77(13):1272–5. Epub 2011/09/16. doi: 10.1212/WNL.0b013e318230208a 21917776

[30] Fischl B. FreeSurfer. Neuroimage. 2012;62(2):774–81. Epub 2012/01/18. doi: 10.1016/j.neuroimage.2012.01.021 22248573 PMC3685476

[31] Wannan CMJ, Cropley VL, Chakravarty MM, Van Rheenen TE, Mancuso S, Bousman C, et al. Hippocampal subfields and visuospatial associative memory across stages of schizophrenia-spectrum disorder. Psychol Med. 2019;49(14):2452–62. Epub 2018/12/05. doi: 10.1017/S0033291718003458 30511607

[32] Malone IB, Leung KK, Clegg S, Barnes J, Whitwell JL, Ashburner J, et al. Accurate automatic estimation of total intracranial volume: a nuisance variable with less nuisance. Neuroimage. 2015;104:366–72. Epub 2014/09/27. doi: 10.1016/j.neuroimage.2014.09.034 25255942 PMC4265726

[33] Desikan RS, Ségonne F, Fischl B, Quinn BT, Dickerson BC, Blacker D, et al. An automated labeling system for subdividing the human cerebral cortex on MRI scans into gyral based regions of interest. Neuroimage. 2006;31(3):968–80. Epub 2006/03/15. doi: 10.1016/j.neuroimage.2006.01.021 16530430

[34] Destrieux C, Fischl B, Dale A, Halgren E. Automatic parcellation of human cortical gyri and sulci using standard anatomical nomenclature. Neuroimage. 2010;53(1):1–15. Epub 2010/06/16. doi: 10.1016/j.neuroimage.2010.06.010 20547229 PMC2937159

[35] Duvernoy O, Malm T, Thuomas KA, Larsson SG, Hansson HE. CT and MR evaluation of pericardial and retrosternal adhesions after cardiac surgery. J Comput Assist Tomogr. 1991;15(4):555–60. Epub 1991/07/01. doi: 10.1097/00004728-199107000-00005 2061466

[36] Talairach J, Tournoux P, Rayport M. Co-planar stereotaxic atlas of the human brain: 3-dimensional proportional system: an approach to cerebral imaging: Thieme Medical Publishers. 1988.

[37] Lamm C, Decety J, Singer T. Meta-analytic evidence for common and distinct neural networks associated with directly experienced pain and empathy for pain. Neuroimage. 2011;54(3):2492–502. Epub 2010/10/16. doi: 10.1016/j.neuroimage.2010.10.014 20946964

[38] Singer T, Seymour B, O’Doherty J, Kaube H, Dolan RJ, Frith CD. Empathy for pain involves the affective but not sensory components of pain. Science. 2004;303(5661):1157–62. Epub 2004/02/21. doi: 10.1126/science.1093535 14976305

[39] Esménio S, Soares JM, Oliveira-Silva P, Gonçalves Ó F, Decety J, Coutinho J. Brain circuits involved in understanding our own and other’s internal states in the context of romantic relationships. Soc Neurosci. 2019;14(6):729–38. Epub 2019/02/27. doi: 10.1080/17470919.2019.1586758 30806571

[40] Hoffmann F, Koehne S, Steinbeis N, Dziobek I, Singer T. Preserved Self-other Distinction During Empathy in Autism is Linked to Network Integrity of Right Supramarginal Gyrus. J Autism Dev Disord. 2016;46(2):637–48. Epub 2015/10/20. doi: 10.1007/s10803-015-2609-0 26476740

[41] Li H, Xue Z, Ellmore TM, Frye RE, Wong ST. Network-based analysis reveals stronger local diffusion-based connectivity and different correlations with oral language skills in brains of children with high functioning autism spectrum disorders. Hum Brain Mapp. 2014;35(2):396–413. Epub 2012/09/26. doi: 10.1002/hbm.22185 23008187 PMC6869619

[42] Reynaud E, Lesourd M, Navarro J, Osiurak F. On the neurocognitive origins of human tool use: A critical review of neuroimaging data. Neurosci Biobehav Rev. 2016;64:421–37. Epub 2016/03/16. doi: 10.1016/j.neubiorev.2016.03.009 26976352

[43] Liégeois F, Mayes A, Morgan A. Neural Correlates of Developmental Speech and Language Disorders: Evidence from Neuroimaging. Curr Dev Disord Rep. 2014;1(3):215–27. Epub 2014/07/25. doi: 10.1007/s40474-014-0019-1 25057455 PMC4104164

[44] Andras N Zsido Nikolett Arato, Ihasz Virag, Basler Julia, Timea Matuz-Budai Orsolya Inhof, et al. "Finding an Emotional Face" Revisited: Differences in Own-Age Bias and the Happiness Superiority Effect in Children and Young Adults. Front Psychol. 2021 Mar 29;12:580565. doi: 10.3389/fpsyg.2021.580565 33854456 PMC8039508

[45] Scherer KR, Clark-Polner E, Mortillaro M. In the eye of the beholder? Universality and cultural specificity in the expression and perception of emotion. Int J Psychol. 2011;46(6):401–35. Epub 2011/12/01. doi: 10.1080/00207594.2011.626049 22126090

[46] Jack RE, Blais C, Scheepers C, Schyns PG, Caldara R. Cultural confusions show that facial expressions are not universal. Curr Biol. 2009;19(18):1543–8. Epub 2009/08/18. doi: 10.1016/j.cub.2009.07.051 19682907

